# Arsenic in Wall-Papers and Dresses

**Published:** 1878-01

**Authors:** 


### Arsenic in Wall-Papers and Dresses.

Of fifty samples of wall-paper recently examined by Professor A. P. Kerley, says the *British Medical Journal*, twelve were found to contain arsenic. The arsenic was present either as arsenite of copper or aceto-arsenite of copper. Two samples, not reported, which contained no green color, were found to contain arsenic; and several with green figures contained no trace of arsenic. Six samples of green tarlatan, all that were tested, were found to contain large amounts of aceto-arsenite of copper. The higher the price paid, the more arsenic was found. The green coloring was held more firmly to the fabric by means of gum arabic and starch. From the results tabulated, it appears that a room sixteen feet square and nine feet high will have spread upon its walls, provided any of these papers are hung, from fifty-two grains to more than eight ounces of green coloring matter. The remedy is simple; be careful not to buy such wall-papers or dresses.—*Med. and Surg. Reporter.*

